# Fibro-Inflammatory Signaling, Fibrosis, and Microvascular Ischemia as Convergent Translational Substrates of Sudden Cardiac Death in Hypertrophic Cardiomyopathy: A Systematic Review

**DOI:** 10.1007/s12265-026-10853-w

**Published:** 2026-10-13

**Authors:** Antonio da Silva Menezes Junior, Isabela Jube Wastowski, Lilian Carla Carneiro

**Affiliations:** 1 Medicine Faculty, Federal University of Goiás, Rua 235 esq. com 5ª Avenida. s/n Setor Universitário, Goiás Goiânia, CEP 74605-020 Brazil; 2 Pontifical Catholic University of Goiás, Goiânia, Goiás Brazil; 3 State University of Goiás, Goiânia, Goiás Brazil; 4 Health Science Post Graduation, Medical College, Federal University of Goiás, Goiânia, Goiás Brazil; 5 Public Health and Tropical Medicine Post Graduation Program, IPTSP—Federal University of Goiás, Goiânia, Brazil

**Keywords:** Hypertrophic cardiomyopathy, Sudden cardiac death, Myocardial fibrosis, Microvascular ischemia, Fibro-inflammatory signaling, Arrhythmogenic substrate

## Abstract

Hypertrophic cardiomyopathy (HCM) remains an important cause of sudden cardiac death (SCD), but the biological determinants of ventricular arrhythmic vulnerability remain incompletely defined. This systematic translational review examined whether fibro-inflammatory signaling, fibrotic remodeling, and microvascular ischemia represent convergent components of the arrhythmogenic substrate in HCM. MEDLINE/PubMed, Embase, Web of Science Core Collection, and Cochrane Library were searched from inception to January 2026. Of 167 records, 37 full-text reports were assessed and 11 studies included. Fibrosis emerged as the most clinically mature substrate marker, while microvascular ischemia appeared to amplify electrical instability, particularly in remodeled myocardium. Molecular and tissue studies suggested upstream fibro-inflammatory programs involving activated fibroblasts, extracellular matrix dysregulation, SMAD/TGF-beta signaling, CRLF1/LIFR-JAK-STAT signaling, and altered multicellular communication. Overall, the evidence supports an integrated mechanism-to-substrate model, but prospective outcome-validated studies are needed before these biological layers can refine SCD risk stratification beyond established clinical and imaging markers in contemporary HCM practice.

**Supplementary Information:**

The online version contains supplementary material available at https://doi.org/10.1007/s12265-026-10853-w.

## Introduction

Hypertrophic cardiomyopathy (HCM) is a common inherited cardiac disorder and an important cause of sudden cardiac death (SCD), particularly in younger and otherwise healthy individuals [1]. Despite advances in imaging, phenotyping, and clinical risk stratification, the prediction of malignant ventricular arrhythmias in HCM remains incomplete [2]. Current risk models are clinically useful but do not fully explain why some patients with apparently intermediate phenotypes develop ventricular tachyarrhythmic events, whereas others with marked structural remodeling remain free of major arrhythmic complications [3]. This discordance suggests that conventional clinical markers do not fully capture the biological substrate underlying ventricular electrical instability in HCM [2, 4].

Among the structural abnormalities implicated in this substrate, myocardial fibrosis is among the most consistently reported [5, 6]. Cardiac magnetic resonance studies have associated fibrosis burden, assessed by late gadolinium enhancement or by fibrosis mass quantification, with adverse outcomes, including sustained ventricular arrhythmias and appropriate implantable cardioverter-defibrillator therapies [4, 7]. Translational studies integrating late gadolinium enhancement, post-contrast T1 mapping, and computational modeling suggest that both dense and diffuse fibrosis may influence arrhythmogenic vulnerability, indicating that the substrate extends beyond conventional focal scar assessment [8, 9]. Together, these observations support fibrosis as a biologically relevant intermediate phenotype within the arrhythmogenic substrate [4, 9].

Fibrosis alone, however, is unlikely to explain the full spectrum of electrical instability in HCM. Evidence also suggests that myocardial ischemia and coronary microvascular dysfunction may modify substrate behavior [10]. Integrated positron emission tomography/cardiac magnetic resonance (PET/CMR) studies have linked fibrosis extension, hypermetabolism, and perfusion abnormalities to higher SCD risk profiles [11]. Translational electrophysiological modeling suggests that ischemic stress may interact with diffuse fibrosis. This interaction may increase substrate heterogeneity, reduce repolarization reserve, and facilitate sustained reentry [12]. Thus, ischemia may function not merely as a consequence of hypertrophy but as a modifier of arrhythmogenic vulnerability [11, 12].

A purely structural model of HCM is also increasingly insufficient. Tissue-based and molecular studies suggest that fibrosis and ischemia may reflect downstream expressions of a broader, biologically active remodeling program rather than isolated end-stage abnormalities [13]. In hypertrophied septal myocardium, dysregulated SMAD signaling has been linked to collagen deposition and profibrotic remodeling [14].

Large myectomy-based analyses have identified fibroblast-derived cytokine receptor-like factor 1 (CRLF1) and downstream leukemia inhibitory factor receptor (LIFR)-JAK1/2-STAT3 signaling as candidate drivers of pathologic remodeling [15]. In parallel, single-nucleus RNA sequencing and spatial transcriptomics have revealed activated fibroblast states, altered immune-cell composition, and profibrotic signaling networks in obstructive HCM tissue [16]. These observations support the hypothesis that fibro-inflammatory signaling may represent an upstream biological layer contributing to overt structural remodeling. This pathway may therefore represent a mechanistic layer in substrate formation [14–16].

Despite growing interest in these mechanisms, the evidence remains fragmented. Available studies span clinical imaging cohorts, biomarker research, histopathologic analyses, and molecular profiling platforms. Clinical investigations have largely focused on fibrosis burden, nonsustained ventricular tachycardia, and imaging-derived risk surrogates [2, 4, 9]. Mechanistic studies have examined fibroblast activation, extracellular matrix signaling, and multicellular remodeling. However, these findings have not been consistently integrated into a unified SCD susceptibility framework [14–16].

Several key gaps remain, particularly the limited outcome-validated evidence directly linking molecular and tissue-level remodeling signatures to adjudicated arrhythmic endpoints [17, 18]. The functional interplay between diffuse fibrosis and microvascular ischemia requires further clarification in larger, prospective cohorts. Limited integration of multimodal data has also constrained the development of substrate-based clinical risk models.

Therefore, this systematic review examined whether fibro-inflammatory signaling, fibrosis, and microvascular ischemia converge as biological components of the ventricular arrhythmogenic substrate underlying SCD susceptibility in HCM. By integrating imaging, tissue-based, and molecular evidence, we aimed to clarify how these domains may interact, evaluate the strength of the evidence, identify key gaps, and outline pathways for future risk refinement.

## Methods

### Study Design

The review followed a predefined protocol registered in PROSPERO (CRD420261350570) and was reported in accordance with the Preferred Reporting Items for Systematic Reviews and Meta-Analyses statement [19]. Given the expected heterogeneity in study designs, biological platforms, phenotype definitions, and endpoint ascertainment, we planned a qualitative synthesis a priori.

### Review Question

The review addressed the following question: What evidence supports fibro-inflammatory signaling, fibrosis, and microvascular ischemia as convergent determinants of the ventricular arrhythmogenic substrate underlying sudden cardiac death in HCM?

For the purposes of this review, fibro-inflammatory signaling was considered the upstream cellular and molecular remodeling layer, fibrosis the structural substrate expression of remodeling, and microvascular ischemia the functional perfusion-related component potentially modifying substrate behavior. These domains were analyzed separately but interpreted in relation to their potential biological convergence within the arrhythmogenic substrate.

### Eligibility Criteria

Studies were eligible if they met the following criteria: (1) original human research; (2) inclusion of patients with hypertrophic cardiomyopathy, including obstructive, nonobstructive, or mixed HCM cohorts; (3) evaluation of at least one prespecified biological domain of interest; and (4) assessment of SCD-related arrhythmic phenotypes, ventricular arrhythmia burden, imaging or histopathologic substrate markers directly relevant to malignant ventricular arrhythmia risk, or mechanistic remodeling elements directly relevant to arrhythmogenic substrate biology.

The prespecified domains were fibro-inflammatory signaling, myocardial fibrosis or extracellular matrix remodeling, coronary microvascular dysfunction or ischemia, and molecular regulatory pathways relevant to substrate formation.

Eligible designs included observational cohort, case-control, and cross-sectional studies; translational imaging investigations; histopathologic analyses; and human molecular or omics-based studies. Reviews, editorials, letters without original data, conference abstracts lacking extractable information, case reports, and very small exploratory case series without an analytical design were excluded. Animal-only studies and in vitro investigations without direct translational relevance to humans were also excluded. Trial design papers and feasibility reports without analyzable biological or clinical outcome data were also excluded.

### Information Sources and Search Strategy

A systematic literature search was performed in MEDLINE/PubMed, Embase, Web of Science Core Collection, and the Cochrane Library from database inception to January 2026. Search strategies combined controlled-vocabulary and free-text terms related to hypertrophic cardiomyopathy, sudden cardiac death, ventricular arrhythmias, fibrosis, extracellular matrix remodeling, coronary microvascular dysfunction, myocardial ischemia and perfusion, fibro-inflammatory or immune remodeling, transcriptomics, and molecular regulatory pathways. The reference lists of eligible studies and relevant review articles were manually screened to identify additional records.

### Study Selection

The relatively small number of included studies reflects the specificity of the predefined eligibility criteria, which required direct evaluation of biologically relevant domains linked to arrhythmogenic substrate formation in hypertrophic cardiomyopathy. In addition, the emerging, highly specialized nature of fibro-inflammatory and molecular research in this field limits the availability of studies that integrate clinical, imaging, and molecular endpoints. Therefore, the final number of included studies should be interpreted as reflecting both methodological rigor and the current stage of evidence development.

All retrieved records were imported into a reference-management platform and deduplicated before screening. Two reviewers (IJW and ASMJ) screened titles and abstracts against the predefined eligibility criteria and then reviewed the full texts of potentially relevant articles. Full-text decisions were based on concordance with the review population, mechanistic domain, and relevance to the arrhythmogenic substrate biology of SCD; disagreements were resolved by a third reviewer (LCC).

### Data Extraction

Data were extracted in a structured format from all included studies. The following variables were collected when available: first author, year of publication, study design, study population, HCM phenotype, sample size, biological domain of interest, method of substrate or phenotypic assessment, arrhythmic or sudden death–related outcomes, major mechanistic findings, and main limitations.

### Methodological Quality and Translational Relevance Assessment

Methodological quality and translational relevance were assessed using prespecified, study-design-specific instruments. Observational cohort, case-control, and analytical cross-sectional studies were appraised using the corresponding Joanna Briggs Institute critical-appraisal checklists [20]. Because several included studies were translational imaging, histopathologic, molecular, spatial transcriptomic, or computational investigations that were not adequately captured by conventional epidemiologic appraisal tools, these studies underwent a prespecified domain-based assessment of methodological quality and translational relevance developed a priori for this review.

Because the included literature was methodologically heterogeneous and no single validated instrument was suitable for all translational study types, these assessments were used to inform the interpretation and weighting of the evidence rather than to derive a pooled summary score. The degree of expected heterogeneity was evaluated qualitatively rather than statistically. Heterogeneity was anticipated across six prespecified domains: study design, HCM phenotype, substrate assessment modality, biological platform, directness of the arrhythmic endpoint, and translational level of inference. Because no quantitative synthesis was performed, statistical heterogeneity metrics, including I², τ², and Cochran’s Q, were not applicable. Detailed criteria for translational studies are provided in the Supplementary Material.

### Data Synthesis

A quantitative meta-analysis was not performed due to substantial heterogeneity across the included studies. This heterogeneity involved differences in study design (observational, translational, and molecular), patient populations (obstructive and nonobstructive phenotypes), substrate assessment methods (imaging, histopathology, and omics-based platforms), and endpoint definitions (ranging from surrogate arrhythmic markers to clinical outcomes). These variations precluded meaningful statistical pooling and justified a qualitative, hierarchical synthesis approach focused on biological integration rather than effect size estimation.

Within this framework, the synthesis examined whether fibro-inflammatory signaling, fibrotic remodeling, and ischemic stress could be interpreted as biologically interrelated components of a unified arrhythmogenic substrate. Greater emphasis was placed on studies providing the most direct linkage between substrate biology and ventricular arrhythmic risk, whereas lower-resolution circulating-biomarker studies were interpreted as supportive and assigned lower evidentiary weight.

## Results

### Search Results and Study Selection

**Fig. 1:**
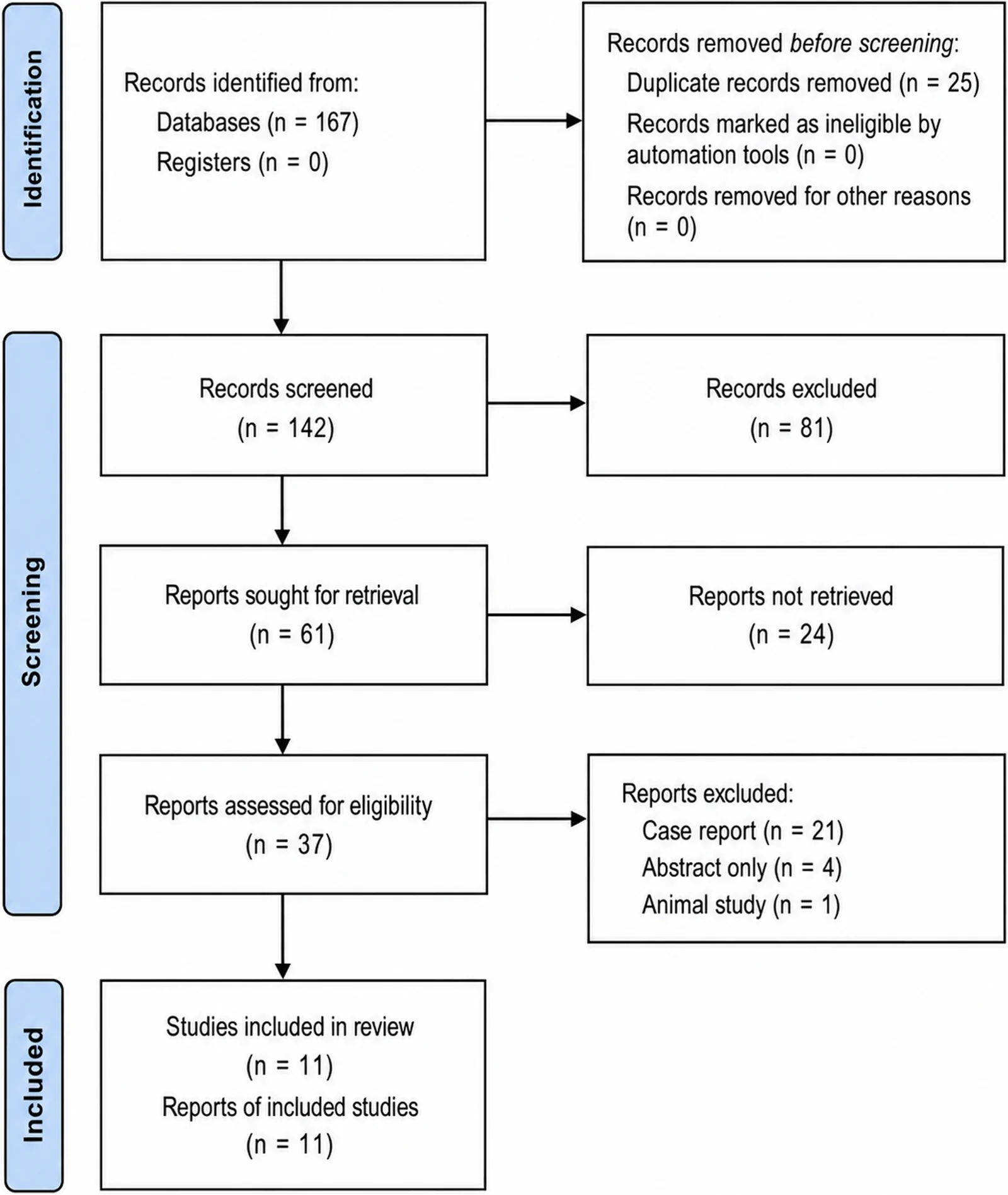
PRISMA 2020 flow diagram of study selection

The search identified 167 records. After removing 25 duplicates, 142 records underwent title and abstract screening. Sixty-one reports were sought for retrieval, of which 37 full-text articles were assessed for eligibility. After exclusion of 26 reports, 11 studies were included in the qualitative synthesis [2, 4, 9, 11–16, 21–25], as shown in Fig. 1. The included studies spanned clinical imaging cohorts, translational tissue analyses, and molecular profiling platforms in HCM. These studies addressed three major biological domains relevant to the arrhythmogenic substrate: fibro-inflammatory signaling, fibrotic remodeling, and microvascular ischemia [2, 4, 9, 11–16, 21, 22, 25]. The study selection process is also summarized in the Supplementary Material. The characteristics of the included studies are summarized in Table 1, and their main findings by arrhythmogenic substrate domain are summarized in Table 2.

**Table 1:** Characteristics of included studies

First author (year)	Study design	HCM phenotype	Sample size	Population	Substrate domain	Endpoint/ substrate outcome
Liu (2024)	Retrospective observational cohort	HOCM	755	Consecutive patients with hypertrophic obstructive cardiomyopathy; LVOT gradient ≥ 30 mm Hg at rest or with provocation	Myocardial injury, fibrosis, ischemia-linked remodeling	NSVT, ventricular ectopy burden, estimated 5-year SCD risk
Lin [15]	Translational tissue-based study	Obstructive HCM	269	Patients undergoing septal myectomy for obstructive HCM	Fibro-inflammatory signaling, cytokine-associated remodeling	Molecular substrate formation; no direct arrhythmic endpoint
Miyaji (2016)	Observational CMR cohort	Mixed HCM	109	Patients with HCM undergoing cardiac magnetic resonance evaluation	Fibrosis burden	Sustained VT/VF, appropriate ICD discharge, composite clinical outcomes
Marques-Alves [11]	Cross-sectional multimodality imaging study	Nonobstructive HCM	30	Adults with sarcomeric nonobstructive HCM evaluated with PET/CMR	Perfusion abnormality, metabolism, fibrosis	SCD risk score, arrhythmia-risk profile
Coleman [12]	Human translational/computational study	HCM	28 imaging patients; human HCM tissue/cellular datasets	Human HCM cardiomyocyte electrophysiology, perfusion imaging, and computational ventricular models	Ischemia, fibrosis, and electrophysiologic remodeling	VT history, inducible reentry, arrhythmogenic behavior
Zhang [14]	Tissue-based immunohistopathologic study	HOCM	17	Pediatric and adult patients with hypertrophic obstructive cardiomyopathy undergoing septal myectomy	Fibrosis signaling	Histopathologic substrate outcome
Liu [23]	Single-nucleus RNA-seq and spatial transcriptomics study	Obstructive HCM	16	Human interventricular septal tissue obtained during surgical myectomy	Fibro-inflammatory signaling, ECM remodeling, multicellular communication	Molecular substrate formation; no direct arrhythmic endpoint
Kunak (2025)	Retrospective observational study	Mixed HCM	124	Patients with obstructive and nonobstructive HCM plus healthy controls	Inflammatory/metabolic biomarker	Cardiovascular death, appropriate ICD shock, composite adverse outcomes in obstructive HCM
Cambronero [22]	Observational biomarker study	Mixed HCM	124	Patients with HCM evaluated for circulating endothelial dysfunction markers	Endothelial/microvascular dysfunction	NSVT, obstruction severity
Sonnenschein [25]	Translational biomarker/tissue study	HCM with HOCM subgroup	60	HCM cohort with serum analysis and myocardial tissue evaluation in HOCM	ECM remodeling, fibrosis biology	Substrate-level remodeling outcome
Shade (2022)	Translational imaging/computational study	HCM	26	Adult patients with HCM, including 13 with clinical ventricular arrhythmias documented by ICD therapy	Dense and diffuse fibrosis	Clinical ventricular arrhythmia outcome; personalized SCD-risk prediction

Abbreviations: *CMR *= cardiac magnetic resonance, *cTnI *= cardiac troponin I, *ECM *= extracellular matrix, *HCM *= hypertrophic cardiomyopathy, *HOCM *= hypertrophic obstructive cardiomyopathy, *ICD* = implantable cardioverter-defibrillator, *LGE* = late gadolinium enhancement, *LVOT* = left ventricular outflow tract, *MMP2* = matrix metalloproteinase-2, *NSVT* = nonsustained ventricular tachycardia, *PET* = positron emission tomography, *SCD* = sudden cardiac death, *VT/VF* = ventricular tachycardia/ventricular fibrillation

**Table 2:** Main findings of the included studies by arrhythmogenic substrate domain

First author, year	Domain	Arrhythmic/SCD relevance	Main finding	Evidentiary contribution
Liu (2024)	Myocardial injury / fibrosis-linked risk	NSVT, ventricular ectopy, estimated 5-year SCD risk	Higher cTnI levels were associated with NSVT, more complex ventricular ectopy, positive LGE, and higher estimated SCD risk in HOCM	High
Lin [15]	Fibro-inflammatory signaling	Indirect substrate relevance	Activated fibroblast-derived CRLF1 promoted hypertrophic remodeling through LIFR-JAK1/2-STAT3 signaling	Moderate
Miyaji (2016)	Fibrosis burden	Sustained VT/VF, appropriate ICD discharge	Greater LV fibrosis mass was associated with poorer clinical outcomes, including malignant ventricular arrhythmias	High
Marques-Alves [11]	Ischemia, metabolism, fibrosis	SCD risk profile	Fibrosis extension, myocardial hypermetabolism, and perfusion abnormalities were associated with higher SCD risk profiles	High
Coleman [12]	Ischemia-fibrosis interaction	VT history, inducible arrhythmogenic behavior	Ischemic stress amplified repolarization abnormalities and interacted with diffuse fibrosis to facilitate sustained re-entry	High
Zhang [14]	Profibrotic signaling	Indirect substrate relevance	Collagen deposition in HOCM was accompanied by increased SMAD2/3 and reduced SMAD7	Moderate
Liu (2023)	Fibroblast activation / multicellular remodeling	Indirect substrate relevance	Activated fibroblast programs, immune remodeling, and spatially organized profibrotic signaling networks characterized fibrotic HCM tissue	Moderate
Kunak (2025)	Inflammatory/metabolic biomarker	Cardiovascular death, appropriate ICD shock, composite adverse outcomes	Uric acid-to-albumin ratio was higher in obstructive HCM and associated with adverse outcomes	Supportive
Cambronero [22]	Endothelial/microvascular dysfunction	NSVT, obstruction severity	von Willebrand factor levels were increased and associated with NSVT and greater obstruction severity	Supportive
Sonnenschein [25]	ECM remodeling/fibrosis biology	Indirect substrate relevance	c-KIT was elevated in serum and myocardial tissue and accompanied by increased Collagen 1A1 and MMP2 expression	Supportive
Shade (2022)	Dense and diffuse fibrosis	Clinical ventricular arrhythmia outcome	Integration of dense and diffuse fibrosis improved model-based prediction of ventricular arrhythmia risk compared with conventional strategies	High

Note: Evidentiary contribution reflects each study’s relative contribution of each study to the qualitative synthesis: high = direct arrhythmic or sudden cardiac death–related relevance with strong translational value; moderate = mechanistic substrate-level relevance without direct hard arrhythmic outcome analysis; supportive = associative or lower-resolution evidence that was directionally consistent but biologically less definitive

### Characteristics of Included Studies

The included studies used diverse methodologies across clinical, translational, and molecular research settings. Clinical and imaging investigations assessed fibrosis burden, myocardial injury, perfusion abnormalities, nonsustained ventricular tachycardia, ventricular arrhythmia events, and SCD risk profiles. These assessments were conducted in cohorts with obstructive, nonobstructive, or mixed HCM [2, 4, 9, 11, 12, 21, 22, 25]. Tissue-based studies, predominantly using septal myectomy specimens from patients with obstructive HCM, reported collagen deposition, extracellular matrix remodeling, and dysregulated profibrotic signaling [14–16, 25]. Molecular and spatial studies identified activated fibroblast programs, cytokine-associated pathways, and altered intercellular communication networks relevant to substrate formation [15, 16]. The included studies were grouped into three evidence layers: molecular and multicellular studies of substrate formation, tissue-level studies of fibrotic remodeling, and clinical/imaging studies linking substrate expression to arrhythmic risk.

### Methodological Quality and Translational Relevance of the Included Studies

Methodological quality varied across the included studies according to study design and level of translational inference. Among the clinical observational studies, most were assessed as having moderate overall concern. This assessment primarily reflected retrospective or cross-sectional designs, single-center recruitment, mixed-phenotype populations, and reliance on surrogate arrhythmic markers, including nonsustained ventricular tachycardia and estimated SCD risk. Few studies focused on well-defined, adjudicated hard arrhythmic outcomes [2, 4, 9, 11, 12, 21, 22]. Studies evaluating fibrosis burden and cardiac magnetic resonance-defined substrate features were generally stronger in exposure assessment than in endpoint directness, whereas circulating-biomarker studies were more vulnerable to confounding and biological nonspecificity [2, 4, 9, 11, 12, 21, 22]. These methodological limitations affected the certainty and generalizability of the synthesis. Heterogeneity in study designs and frequent reliance on surrogate endpoints limited the certainty of the inferences from this evidence base, as shown in Table 3.

**Table 3:** Risk-of-bias and methodological-quality assessment of included studies Section A. Clinical observational studies

Section A. Clinical observational studies									
First author, year	Study design	JBI tool used	Key concerns	Overall concern					
Liu (2024)	Retrospective cohort	JBI Cohort Checklist	Retrospective single-center design; NSVT and estimated SCD risk used instead of hard SCD outcomes	Moderate					
Miyaji (2016)	Observational cohort	JBI Cohort Checklist	Mixed HCM cohort; composite endpoint includes arrhythmic and non-arrhythmic events	Moderate					
Marques-Alves [11]	Cross-sectional imaging study	JBI Analytical Cross-Sectional Checklist	SCD risk profile used rather than hard arrhythmic outcomes	Moderate					
Kunak (2025)	Retrospective cohort	JBI Cohort Checklist	Composite outcome; indirect biomarker; retrospective design	Moderate to high					
Cambronero [22]	Cross-sectional biomarker study	JBI Analytical Cross-Sectional Checklist	Mixed HCM cohort; NSVT and obstruction severity used as surrogate markers	Moderate					
Section B. Translational imaging, tissue, molecular, and computational studies									
First author, year	Study type	Population/phenotype	Substrate measurement	Outcome directness		Analysis/validation		Overall methodological concern	
Shade (2022)	Translational imaging/computational study	Clear adult HCM cohort	LGE + T1-based substrate definition		Moderate to high		Computational validation against clinical VA		Moderate
Coleman [12]	Translational electrophysiologic/computational study	Human HCM-informed datasets	Strong perfusion/electrophysiologic substrate modeling		Moderate		Good analytic framework, but model-dependent		Moderate
Zhang [14]	Tissue-based histopathologic study	Clear HOCM myectomy cohort	Strong tissue fibrosis signaling assessment		Indirect		Laboratory methods reported; limited sample size		Moderate
Lin [15]	Tissue-based molecular study	Strong obstructive HCM myectomy cohort	Strong RNA-seq/single-cell/functional validation		Indirect		Multimodal validation performed		Moderate
Liu (2023)	Single-nucleus RNA-seq/spatial transcriptomics study	Clear obstructive HCM myectomy cohort	Strong spatial and transcriptomic substrate definition		Indirect		Strong analytic depth; no direct arrhythmic validation		Moderate
Sonnenschein [25]	Translational biomarker/tissue study	Acceptable phenotype definition	Moderate ECM/fibrosis-related assessment		Indirect		Acceptable tissue plus in vitro support		Moderate

Abbreviation: *VA* = ventricular arrhythmia. Overall concern was assessed qualitatively as low, moderate, moderate-to-high, or high based on study-design–specific risk-of-bias features, endpoint directness, exposure/substrate measurement, confounding, analytic transparency, and validation strategy. *JBI* Joanna Briggs Institute, *HCM*, hypertrophic cardiomyopathy, *HOCM* hypertrophic obstructive cardiomyopathy, *LGE* late gadolinium enhancement, *NSVT* nonsustained ventricular tachycardia, *RNA*-seq, *RNA* sequencing, *SCD* sudden cardiac death, *VA* ventricular arrhythmia"

Among translational imaging, tissue, molecular, and computational studies, overall methodological concern was also most commonly moderate. These studies were generally stronger in phenotype definition, substrate characterization, and analytical depth, particularly when multimodal valdation or functional experiments were available [9, 12–16, 25]. However, most were limited by indirect linkage to clinical arrhythmic outcomes, relatively small tissue cohorts, or dependence on mechanistic, histopathologic, or model-based endpoints rather than prospective ventricular arrhythmia events. Computational and spatial studies primarily provided mechanistic information on substrate biology rather than direct prognostic evidence [3, 4, 9, 12–16].

Overall, the methodological quality assessment indicated that the evidence base was heterogeneous and did not include a single uniformly high-certainty study design. In hierarchical, domain-based synthesis, clinical imaging studies were assigned greater weight for direct linkage to arrhythmic risk and patient outcomes, whereas tissue-based and molecular studies were assessed primarily for mechanistic insight into substrate biology. Evidence from studies using surrogate or indirect endpoints was treated as supportive and assigned a lower weight in the overall synthesis. Clinical imaging studies provided the most direct arrhythmic relevance, whereas tissue-based and molecular studies provided greater mechanistic depth.

### Fibro-Inflammatory and Molecular Pathways Underlying Substrate Formation

The included molecular and tissue-based studies suggested that arrhythmogenic substrate formation in HCM may involve a fibro-inflammatory remodeling program, although direct validation against hard arrhythmic outcomes remains limited.

Single-nucleus RNA sequencing and spatial transcriptomic analyses of human obstructive HCM tissue revealed that fibrotic regions were embedded within a multicellular remodeling network characterized by activated fibroblast states, altered immune cell composition, and dysregulated intercellular communication [16]. Profibrotic fibroblast programs were enriched in spatially defined fibrotic and perifibrotic regions, whereas candidate regulators, including AEBP1, RUNX1, MEOX1, and transforming growth factor-β-associated pathways, were implicated in extracellular matrix remodeling and fibroblast activation [16]. These findings indicated that fibrosis in HCM was associated with a coordinated signaling environment involving fibroblasts, immune cells, and cardiomyocytes [16].

Together, the molecular and spatial studies suggested an association between structural remodeling and upstream fibro-inflammatory processes in HCM. Within this framework, fibroblast activation, cytokine-associated signaling, and altered multicellular communication were identified as candidate upstream processes linked to tissue- and imaging-level substrate features [15, 16].

### Fibrotic Remodeling as a Structural Expression of the Arrhythmogenic Substrate

Tissue-based and imaging studies described profibrotic remodeling in HCM, particularly in obstructive phenotypes. In septal myocardium from patients with hypertrophic obstructive cardiomyopathy undergoing myectomy, immunohistopathologic analysis showed marked collagen deposition, increased SMAD2 and SMAD3 expression, and reduced SMAD7 expression, consistent with activation of canonical profibrotic signaling pathways [14]. In a complementary translational study, myocardial c-KIT overexpression in hypertrophic obstructive cardiomyopathy was accompanied by increased expression of collagen 1A1 and matrix metalloproteinase-2 [25].

At the clinical imaging level, fibrosis was a recurrent correlate of arrhythmia-prone phenotypes. In a cardiac magnetic resonance-based cohort, greater left ventricular fibrosis mass was associated with poorer clinical outcomes, including sustained ventricular tachycardia or ventricular fibrillation and appropriate implantable cardioverter-defibrillator discharge [4]. A similar association was observed in obstructive hypertrophic cardiomyopathy, in which elevated cardiac troponin I was associated with nonsustained ventricular tachycardia, more complex ventricular ectopy, positive late gadolinium enhancement, and higher estimated 5-year SCD risk [21]. Although troponin is not a fibrosis marker per se, its association with structural and arrhythmic features suggested a possible injury-fibrosis continuum in higher-risk phenotypes [21].

Fibrosis-related risk also extended beyond focal scar visualization alone. In a translational study integrating late gadolinium enhancement cardiac magnetic resonance, post-contrast T1 mapping, and individual-specific computational heart modeling, dense and diffuse fibrosis were incorporated into personalized ventricular substrates and evaluated against clinical outcomes for ventricular arrhythmia [9]. Compared with models based solely on late gadolinium enhancement, the combined late gadolinium enhancement–T1 approach yielded a more extensive representation of fibrotic remodeling, increased in silico arrhythmia inducibility, and higher predictive performance for clinical ventricular arrhythmia events than conventional guideline-based risk strategies [9]. These data suggested that diffuse fibrosis may increase the complexity of the arrhythmogenic substrate and that conventional structural assessment may underestimate the electrical consequences of nonfocal remodeling [9].

Overall, these studies identified fibrosis as a structural feature of a broader remodeling process in HCM. The available evidence suggested that both focal and diffuse fibrosis may contribute to arrhythmogenic substrate formation and that the clinical relevance of fibrosis may depend on its burden, architecture, and integration within the ventricular myocardium [4, 9, 14, 21, 25].

### Microvascular Ischemia as a Functional Amplifier of Arrhythmogenic Vulnerability

Multimodality imaging and translational electrophysiological studies provided evidence for the ischemic dimension of substrate behavior. In nonobstructive HCM, integrated PET/CMR analysis showed that fibrosis extension, myocardial hypermetabolism, and perfusion abnormalities were associated with higher SCD risk profiles [11]. These findings indicated that metabolic stress and perfusion impairment coexisted with structural remodeling in higher-risk phenotypes [11].

Translational work combining human cardiomyocyte electrophysiology, perfusion imaging, and computer modeling suggested that ischemic stress can amplify repolarization abnormalities and interact with diffuse fibrosis to facilitate sustained ventricular reentry [12]. Within that model, fibrosis and ischemia interacted to determine arrhythmogenic behavior [12]. Supportive biomarker evidence was directionally consistent with these findings: plasma von Willebrand factor, a marker of endothelial dysfunction, was increased in HCM and was associated with nonsustained ventricular tachycardia and greater obstruction severity [22].

Overall, the available evidence suggests that microvascular ischemia may function as a dynamic amplifier of arrhythmogenic vulnerability in HCM, particularly when superimposed on fibrotic myocardial remodeling. Together with the fibrosis-focused studies, these data were consistent with a substrate model in which structural and ischemic remodeling are interrelated [11, 12, 22].

### Integrated Translational Model of the Arrhythmogenic Substrate

Taken together, the included studies were consistent with a hierarchical model in which SCD susceptibility in HCM involves the convergence of fibro-inflammatory signaling, fibrotic remodeling, and microvascular ischemia [4, 9, 11–16, 21–23]. In this framework, activated fibroblasts, cytokine-associated pathways, and altered multicellular communication were categorized as upstream processes; fibrosis and ischemia were categorized as intermediate structural and functional substrate expressions; and ventricular arrhythmias were considered downstream clinical manifestations of integrated remodeling.

Clinical and imaging studies showed that fibrosis burden, myocardial injury, perfusion abnormalities, and ischemia-sensitive electrophysiological changes were associated with ventricular arrhythmic phenotypes or higher SCD risk [4, 9, 11, 12, 21, 22]. Tissue-based and molecular studies identified potential mechanisms for these substrate expressions, including active profibrotic signaling, extracellular matrix dysregulation, fibroblast-derived paracrine mediators, and altered multicellular communication [14–16, 25].

Overall, the literature was consistent with an integrated pathobiological model in which SCD susceptibility in HCM may reflect convergent remodeling biology rather than isolated structural change [4, 9, 11–25].

## Discussion

### Principal Findings

This systematic translational review supports a hierarchical mechanism-to-substrate model of SCD susceptibility in HCM, in which fibro-inflammatory signaling, fibrotic remodeling, and microvascular ischemia are biologically connected but supported by different levels of evidence. Across the included studies, activated fibroblast programs, cytokine-associated pathways, and altered intercellular communication emerged as plausible upstream processes [14–16, 25]; fibrosis represented the most clinically mature structural substrate expression [4, 9, 21, 26–31]; and microvascular ischemia appeared to act as a functional modifier capable of increasing electrical instability, particularly in remodeled myocardium [11, 12, 27]. Ventricular arrhythmias therefore represent the downstream clinical expression of an integrated substrate in which molecular signaling, tissue architecture, and perfusion abnormalities may interact to shape arrhythmic vulnerability [2–28].

### Fibrosis as the Most Clinically Mature Substrate Marker

Among the three domains, fibrosis remains the most clinically mature substrate marker. In this synthesis, imaging studies linked fibrosis burden to sustained ventricular arrhythmias, device therapies, and higher arrhythmic risk profiles [4, 9, 21, 30]. External evidence supports this association. Larger cohorts have shown that greater late gadolinium enhancement burden is associated with SCD or aborted SCD and may improve risk discrimination beyond conventional clinical markers [26, 31]. Additional work suggests that fibrosis distribution may further refine prognostic interpretation [28]. Together, these observations support fibrosis as the strongest current structural correlate of arrhythmogenic risk in HCM [4, 9, 21, 26, 28, 31].

At the same time, the fibrosis literature highlights a central limitation: fibrosis quantification remains methodologically variable [29]. The presence, burden, distribution, and indices of diffuse fibrosis on late gadolinium enhancement are often treated as interchangeable, although they are likely to reflect distinct biological layers [4, 9, 26, 28]. The study by O’Hara et al. linked the electrical impact of fibrosis to both its extent and structural characteristics [9]. By combining late gadolinium enhancement with T1-derived diffuse fibrosis in personalized computational models, this study correlated modeled substrate features with clinical outcomes of ventricular arrhythmia.

Thus, the fibrosis literature suggests that arrhythmogenic risk may depend not only on scar burden but also on the organization of fibrotic remodeling [9, 28]. Future studies should therefore prioritize standardized fibrosis quantification and reporting. These strategies could include consensus protocols for late gadolinium enhancement thresholding, uniform reporting of focal and diffuse fibrosis using validated imaging criteria, and reproducible multicenter measurement techniques. Core laboratory analysis, clearer definitions of diffuse versus focal fibrosis, and continuous reporting of fibrosis pattern and burden may help reduce methodological variability. These steps may enhance reproducibility and facilitate cross-study comparisons.

### Microvascular Ischemia and Substrate Instability

A second implication is that microvascular ischemia may be underweighted in contemporary HCM risk frameworks. In the studies included, perfusion abnormalities, hypermetabolism, and ischemia-sensitive electrophysiological changes were associated with higher-risk substrate profiles and greater arrhythmogenic vulnerability [11, 12]. This interpretation is supported by external evidence showing that coronary microvascular dysfunction is an independent predictor of clinical deterioration and death in HCM [27]. These data argue against viewing ischemia solely as a by-product of hypertrophy. Rather, ischemia may function as a dynamic amplifier of substrate instability, particularly when interacting with diffuse fibrotic remodeling [11, 12, 27].

The ischemia–fibrosis interaction may therefore represent an important translational insight. Fibrosis appears to provide the more established structural scaffold of the arrhythmogenic substrate, whereas microvascular ischemia may act as a functional modifier that increases electrical instability within already remodeled myocardium [4, 9, 11, 12, 27]. The available evidence does not establish ischemia as a fibrosis-independent causal pathway to ventricular arrhythmias; rather, it supports an interaction model in which perfusion impairment may amplify the arrhythmogenic consequences of structural remodeling. This distinction preserves microvascular ischemia as a biologically relevant domain while recognizing that its strongest current clinical significance lies in its interaction with fibrotic substrate rather than as a standalone risk marker.

### Fibro-Inflammatory Signaling and Molecular Substrate Formation

The fibro-inflammatory and molecular signaling domain is a major translational component of this synthesis and is particularly relevant to basic cardiovascular research. The included CRLF1, SMAD, spatial transcriptomic, and extracellular matrix studies suggest that fibrosis in HCM is not merely accumulated collagen but rather reflects an active, multicellular remodeling program involving activated fibroblasts, cytokine-associated signaling, extracellular matrix regulation, and altered cell-cell communication [13–16, 25]. Spatial and single-nucleus transcriptomic data further suggest altered immune-cell composition and immune–stromal interactions within fibrotic HCM tissue [13, 16], supporting immune remodeling as a plausible component of this upstream biological layer. However, these data do not yet define a reproducible immune-cell profile associated with ventricular arrhythmic risk. Thus, the current evidence identifies plausible molecular and multicellular processes contributing to substrate formation, while their direct prognostic relevance remains unproven.

However, this domain is also the most vulnerable to overinterpretation. Most molecular and transcriptomic studies did not report adjudicated outcomes for SCD, ventricular tachycardia/ventricular fibrillation, or appropriate implantable cardioverter-defibrillator therapy [13–16, 25]. Their contribution is therefore primarily explanatory rather than predictive. They clarify how the substrate may form, but not how these signals should be used clinically. The translational gap between mechanistic insight and clinically validated arrhythmic outcomes is therefore a central limitation of the current evidence base. Bridging this gap will require prospective studies integrating molecular profiling with standardized imaging and well-defined clinical endpoints [13–16, 25].

### Genotype–Phenotype Interaction and Substrate Vulnerability

Hypertrophic cardiomyopathy is primarily a genetic myocardial disease, most often driven by pathogenic or likely pathogenic variants in sarcomeric genes, and the biological substrate described in this review is therefore unlikely to be genotype-neutral. Earlier SHaRe registry data established that sarcomeric HCM is associated with a greater lifetime burden of disease and adverse outcomes [32]. More recent SHaRe data extend this observation by showing that sarcomeric and nonsarcomeric HCM also differ in longitudinal disease trajectory, comorbidity profile, ventricular arrhythmia incidence, and HCM-related mortality [33]. These findings suggest that sarcomeric mutations may define an upstream myocardial vulnerability through altered mechanics, energetics, calcium handling, and mechanotransduction, while fibro-inflammatory activation, extracellular matrix remodeling, fibrosis, microvascular ischemia, and arrhythmic susceptibility may represent downstream expressions whose relative contribution differs across genetic subgroups. However, current evidence remains insufficient to define genotype-specific fibro-inflammatory or ischemic signatures. Future studies should therefore integrate sarcomeric mutation status with advanced imaging, spatial transcriptomics, perfusion assessment, electrophysiological substrate characterization, and adjudicated ventricular arrhythmic outcomes.

### Electrical Substrate Assessment and the Missing Electrophysiological bridge

A major translational gap is the limited integration between biological substrate markers and direct electrophysiological characterization in HCM. Most available studies infer arrhythmogenicity from CMR-defined fibrosis, histopathology, molecular signatures, NSVT, ICD therapies, or computational modeling, but these approaches do not fully define the human ventricular electrical substrate. Invasive electroanatomic mapping, high-density voltage mapping, assessment of conduction delay, fractionated electrograms, and programmed ventricular stimulation may help determine whether fibrotic, ischemic, or fibro-inflammatory regions correspond to low-voltage myocardium, slow conduction, abnormal electrograms, or reentry-prone tissue. Future studies should combine genotype, CMR, perfusion imaging, molecular biomarkers, computational modeling, and invasive or device-derived electrophysiological data to determine whether multimodal substrate markers identify truly arrhythmogenic myocardium [34–37]. 

### Circulating Biomarkers and Evidentiary Caution

The circulating-biomarker literature requires greater caution. Cardiac troponin I appears to be the strongest circulating marker in this evidence base because it has been associated with nonsustained ventricular tachycardia, late gadolinium enhancement, and estimated 5-year SCD risk in a large cohort of patients with obstructive HCM [21]. By contrast, the uric acid-to-albumin ratio and von Willebrand factor are less biologically specific and may reflect downstream inflammatory, endothelial, or injury-related responses [22, 24]. These markers support the interpretation that high-risk HCM may involve active remodeling, but they do not define the substrate with the same precision as imaging, histopathology, or tissue-level molecular studies [21, 22, 24].

### Evidence Quality and Translational Implications

From an evidence-quality perspective, the field is uneven but convergent. The fibrosis domain has the strongest human outcome linkage and the highest current translational confidence [4, 9, 21, 26, 28]. The ischemia/microvascular domain is biologically compelling and supported by prognostic evidence, yet remains underrepresented in formal clinical risk frameworks [11, 12, 22, 27]. The fibro-inflammatory domain is mechanistically rich but currently has the lowest level of direct clinical certainty because its signals remain largely unanchored to hard arrhythmic outcomes [14–16, 25]. This asymmetry is a central finding of the review [2, 4, 9, 11, 12, 21, 22].

These observations have implications for future research. Future work should move beyond isolated biomarkers toward integrated substrate models. A next-generation HCM framework will likely need to combine (1) structural burden, including focal and diffuse fibrosis; (2) microvascular or perfusion abnormalities; and (3) selected mechanistically grounded molecular signals that are reproducible, externally validated, and prospectively linked to outcomes [4, 14–16]. Current guidelines appropriately remain conservative and continue to prioritize established clinical and imaging markers [2]. Future refinement of arrhythmic risk assessment may therefore depend less on isolated clinical variables and more on biologically integrated models that account for interactions among fibro-inflammatory signaling, fibrosis, and ischemia [2, 4, 9, 11, 12, 21, 22].

### Therapeutic Implications: from Substrate Identification to Substrate Modification

The therapeutic implications of this integrated model remain hypothesis-generating. Current prevention of sudden cardiac death in HCM is still based mainly on clinical risk stratification and ICD therapy rather than direct modification of the ventricular arrhythmogenic substrate. However, if fibro-inflammatory signaling, fibrosis, and microvascular ischemia are confirmed as biologically connected drivers of electrical vulnerability, future strategies may shift from substrate detection toward substrate modification. Antifibrotic approaches, cytokine- or inflammation-targeted interventions, therapies that improve microvascular function or myocardial energetics, and disease-modifying sarcomere-directed drugs may become relevant if they demonstrate effects on remodeling and on arrhythmic outcomes. Future trials should therefore incorporate adjudicated arrhythmic endpoints, quantitative fibrosis assessment, perfusion imaging, and biomarker profiling to determine whether modification of the biological substrate translates into meaningful risk reduction. At present, however, no antifibrotic, anti-inflammatory, metabolic, or microvascular-targeted therapy has been validated specifically to reduce SCD risk in HCM through substrate modification [2, 38–47].

### Future Directions

Several research directions may advance this integrated approach. Prospective multicenter cohort studies are needed to validate substrate-based risk models that combine advanced imaging, such as quantitative late gadolinium enhancement and T1 mapping, perfusion assessment, including positron emission tomography or stress cardiac magnetic resonance, and multiplexed molecular profiling. Embedding multi-omics platforms, such as single-cell transcriptomics, proteomics, and spatial analyses, into clinical studies may link upstream molecular states with imaging-defined substrate features and clinical outcomes. Randomized or observational studies should incorporate systematic biobanking of blood and tissue samples for longitudinal molecular phenotyping. Where possible, cross-disciplinary collaborations should use computational modeling to simulate substrate behavior using individualized multimodal data. These approaches may clarify the temporal and functional interplay among fibrosis, ischemia, and fibro-inflammatory signaling. Prospective validation of composite risk algorithms integrating structural, functional, and molecular data will be critical before these insights can be translated into risk stratification or targeted preventive interventions in HCM.

### Limitations

This review has several limitations. The included studies were methodologically heterogeneous, ranging from observational cohorts to computational imaging and molecular tissue analyses; therefore, quantitative pooling was not appropriate [2–14]. Endpoints were not uniform; some studies focused on sustained ventricular arrhythmias or device therapies, whereas others relied on nonsustained ventricular tachycardia, estimated SCD risk, or mechanistic substrate surrogates, limiting the extent to which upstream molecular and tissue-level findings can currently be translated into routine clinical decision-making [2–5, 10, 11, 14]. Several informative mechanistic studies were based on myectomy tissue and were therefore enriched for obstructive or advanced phenotypes, which may limit their applicability across the broader HCM spectrum [6–8, 14]. In particular, the predominance of obstructive HCM cases in tissue-based studies limits the generalizability of detailed mechanistic insights to nonobstructive HCM. Pediatric populations were not systematically represented; therefore, extrapolation to younger patients or early-onset disease should be made with caution. Finally, because this review adopted a translational scope, it integrated evidence with different levels of clinical directness. This approach broadens biological insight but increases heterogeneity in inference [2–17].

### Overall Synthesis / Clinical Relevance

Overall, the available literature supports a model in which SCD susceptibility in HCM may reflect the convergence of fibro-inflammatory signaling, fibrotic remodeling, and microvascular ischemia rather than structural abnormalities alone [2, 4, 9, 11–17]. The field has therefore moved beyond a purely morphologic understanding of HCM but has not yet reached the stage at which molecular substrate markers can be routinely incorporated into clinical risk stratification [2, 14–16, 25]. Clinically, this framework suggests that future risk assessment may benefit from integrating structural fibrosis, perfusion abnormalities, and biologically grounded molecular information rather than relying on isolated substrate markers. However, these molecular and multimodal approaches remain investigational and require prospective validation against adjudicated ventricular arrhythmic outcomes before they can modify current guideline-based practice. The next challenge is therefore to merge advanced imaging, perfusion biology, and mechanistically grounded molecular signals into outcome-validated models capable of refining biologically informed SCD risk [9, 26–28].

## Conclusion

In this systematic translational review, susceptibility to sudden cardiac death in hypertrophic cardiomyopathy was best explained by an integrated substrate model in which fibro-inflammatory signaling, fibrotic remodeling, and microvascular ischemia interact rather than operate in isolation. Within this framework, fibro-inflammatory signaling represents a plausible upstream remodeling process, fibrosis remains the most clinically established structural marker of arrhythmic risk, and microvascular dysfunction may act as a functional amplifier of electrical instability. Molecular and fibro-inflammatory signatures provide important mechanistic insight into substrate formation, but their clinical use remains limited by the absence of prospective outcome validation. Future risk refinement will require multimodal models integrating standardized fibrosis quantification, perfusion assessment, and reproducible molecular markers with adjudicated arrhythmic outcomes.

## Supplementary Information

Below is the link to the electronic supplementary material.

Supplementary Material 1 (DOCX 1.40 MB)

## Data Availability

All data generated or analyzed during this review are included in this article and its supplementary material. No analytic code was generated.
