# Supplementary material for: Fibro-Inflammatory Signaling, Fibrosis, and Microvascular Ischemia as Convergent Translational Substrates of Sudden Cardiac Death in Hypertrophic Cardiomyopathy: A Systematic Review
Source: J Cardiovasc Transl Res. 2026 Oct 13;19(1):133. doi: 10.1007/s12265-026-10853-w (PMC13661659; doi:10.1007/s12265-026-10853-w)
Supplement: Supplementary file 1 [file 12265_2026_10853_MOESM1_ESM.docx]

**Supplementary Material**

*Fibro-Inflammatory Signaling, Fibrosis, and Microvascular Ischemia in the Arrhythmogenic Substrate of Sudden Cardiac Death in Hypertrophic Cardiomyopathy: A Systematic Review*

**Contents**

- Supplementary Figure 1. PRISMA 2020 flow diagram
- Supplementary Table S1. Eligibility decisions for studies assessed at full-text review
- Supplementary Table S2. Full search strategies
- Supplementary Table S3. Methodological quality and translational relevance assessment of the included studies
- Supplementary Appendix 1. Domain-based methodological quality framework for translational studies
- Supplementary Appendix 2. Abbreviations

*Note: Page numbers refer to the main manuscript and exclude the title page. “Suppl.” refers to the Supplementary Material file.*

| **Section and Topic** | **Item #** | **Checklist item** | **Location where the item is reported** |
| --- | --- | --- | --- |
| **TITLE** | | |  |
| Title | 1 | Identify the report as a systematic review. | Title page |
| **ABSTRACT** | | |  |
| Abstract | 2 | See the PRISMA 2020 for Abstracts checklist. | p. 1 |
| **INTRODUCTION** | | |  |
| Rationale | 3 | Describe the rationale for the review in the context of existing knowledge. | pp. 1-3 |
| Objectives | 4 | Provide an explicit statement of the objective(s) or question(s) the review addresses. | p. 3 |
| **METHODS** | | |  |
| Eligibility criteria | 5 | Specify the inclusion and exclusion criteria for the review and how studies were grouped for the syntheses. | pp. 3-4 |
| Information sources | 6 | Specify all databases, registers, websites, organizations, reference lists, and other sources searched or consulted to identify studies. Specify the date when each source was last searched or consulted. | p. 4; Suppl., pp. 3-5 |
| Search strategy | 7 | Present the full search strategies for all databases, registers, and websites, including any filters and limits used. | Suppl., pp. 3-5 |
| Selection process | 8 | Specify the methods used to decide whether a study met the inclusion criteria of the review, including how many reviewers screened each record and each report retrieved, whether they worked independently, and, if applicable, details of automation tools used in the process. | pp. 4-5 |
| Data collection process | 9 | Specify the methods used to collect data from reports, including how many reviewers collected data from each report, whether they worked independently, any processes for obtaining or confirming data from study investigators, and if applicable, details of automation tools used in the process. | p. 5 |
| Data items | 10a | List and define all outcomes for which data were sought. Specify whether all results that were compatible with each outcome domain in each study were sought (e.g., for all measures, time points, analyses), and if not, the methods used to decide which results to collect. | p. 5; Table 2 |
|  | 10b | List and define all other variables for which data were sought (e.g., participant and intervention characteristics, funding sources). Describe any assumptions made about any missing or unclear information. | p. 5; Table 1 |
| Study risk of bias assessment | 11 | Specify the methods used to assess risk of bias in the included studies, including details of the tool(s) used, how many reviewers assessed each study and whether they worked independently, and if applicable, details of automation tools used in the process. | p. 5; Table 3; Suppl., pp. 6-7 |
| Effect measures | 12 | Specify for each outcome the effect measure(s) (e.g. risk ratio, mean difference) used in the synthesis or presentation of results. | p. 5 (N/A) |
| Synthesis methods | 13a | Describe the processes used to decide which studies were eligible for each synthesis (e.g. tabulating the study intervention characteristics and comparing against the planned groups for each synthesis (item #5)). | pp. 5-6; Tables 1-2 |
|  | 13b | Describe any methods required to prepare the data for presentation or synthesis, such as handling of missing summary statistics, or data conversions. | p. 5 (N/A) |
|  | 13c | Describe any methods used to tabulate or visually display results of individual studies and syntheses. | p. 6; Tables 1-3; Suppl., pp. 1-7 |
|  | 13d | Describe any methods used to synthesize results and provide a rationale for the choice(s). If meta-analysis was performed, describe the model(s), method(s) to identify the presence and extent of statistical heterogeneity, and software package(s) used. | p. 5 |
|  | 13e | Describe any methods used to explore possible causes of heterogeneity among study results (e.g., subgroup analysis, meta-regression). | p. 5 (N/A) |
|  | 13f | Describe any sensitivity analyses conducted to assess the robustness of the synthesized results. | p. 5 (N/A) |
| Reporting bias assessment | 14 | Describe any methods used to assess the risk of bias due to missing results in a synthesis (arising from reporting biases). | p. 5 (N/A) |
| Certainty assessment | 15 | Describe any methods used to assess certainty (or confidence) in the body of evidence for an outcome. | p. 5; Table 3 |
| **RESULTS** | | |  |
| Study selection | 16a | Describe the results of the search and selection process, from the number of records identified in the search to the number of studies included in the review, ideally using a flow diagram. | p. 6; Fig. 1; Suppl., p. 1 |
|  | 16b | Cite studies that might appear to meet the inclusion criteria, but which were excluded, and explain why they were excluded. | Suppl., pp. 1-2, Table S1 |
| Study characteristics | 17 | Cite each included study and present its characteristics. | p. 6; Table 1 |
| Risk of bias in studies | 18 | Present assessments of risk of bias for each included study. | pp. 6-7; Table 3; Suppl., pp. 6-7 |
| Results of individual studies | 19 | For all outcomes, present, for each study: (a) summary statistics for each group (where appropriate) and (b) an effect estimate and its precision (e.g., confidence/credible interval), ideally using structured tables or plots. | pp. 7-9; Table 2 |
| Results of syntheses | 20a | For each synthesis, briefly summarise the characteristics and risk of bias among contributing studies. | pp. 6-9; Table 3 |
|  | 20b | Present the results of all statistical syntheses conducted. If meta-analysis was done, present for each the summary estimate and its precision (e.g., confidence/credible interval) and measures of statistical heterogeneity. If comparing groups, describe the direction of the effect. | p. 5 (N/A) |
|  | 20c | Present the results of all investigations of possible causes of heterogeneity among study results. | pp. 5, 13 (qualitative) |
|  | 20d | Present the results of all sensitivity analyses conducted to assess the robustness of the synthesized results. | p. 5 (N/A) |
| Reporting biases | 21 | Present assessments of risk of bias due to missing results (arising from reporting biases) for each synthesis assessed. | p. 5 (N/A) |
| Certainty of evidence | 22 | Present assessments of certainty (or confidence) in the body of evidence for each outcome assessed. | p. 5; pp. 12-13; Table 3 |
| **DISCUSSION** | | |  |
| Discussion | 23a | Provide a general interpretation of the results in the context of other evidence. | pp. 9-12 |
|  | 23b | Discuss any limitations of the evidence included in the review. | pp. 12-13 |
|  | 23c | Discuss any limitations of the review processes used. | p. 13 |
|  | 23d | Discuss implications of the results for practice, policy, and future research. | pp. 12-14 |
| **OTHER INFORMATION** | | |  |
| Registration and protocol | 24a | Provide registration information for the review, including register name and registration number, or state that the review was not registered. | p. 3 |
|  | 24b | Indicate where the review protocol can be accessed, or state that a protocol was not prepared. | p. 3 |
|  | 24c | Describe and explain any amendments to information provided at registration or in the protocol. | p. 3 (N/A) |
| Support | 25 | Describe sources of financial or non-financial support for the review, and the role of the funders or sponsors in the review. | p. 14 |
| Competing interests | 26 | Declare any competing interests of review authors. | p. 14 |
| Availability of data, code, and other materials | 27 | Report which of the following are publicly available and where they can be found: template data collection forms; data extracted from included studies; data used for all analyses; analytic code; any other materials used in the review. | p. 14; Suppl., pp. 1-7 |

*From:*  Page MJ, McKenzie JE, Bossuyt PM, Boutron I, Hoffmann TC, Mulrow CD, et al. The PRISMA 2020 statement: an updated guideline for reporting systematic reviews. BMJ 2021;372:n71. doi: 10.1136/bmj.n71. This work is licensed under CC BY 4.0. To view a copy of this license, visit <https://creativecommons.org/licenses/by/4.0/>

**Supplementary Figure 1. PRISMA 2020 Flow Diagram**

**Supplementary Table S1. Eligibility Decisions for Studies Assessed at Full-Text Review**

| **First author, year** | **HCM phenotype** | **Main domain** | **Decision** | **Reason for inclusion or exclusion** |
| --- | --- | --- | --- | --- |
| Kunak, 2025 | Mixed HCM (obstructive and nonobstructive) | Inflammatory/metabolic circulating biomarker | Include | Original human HCM study with clinically relevant outcomes, including cardiovascular death and appropriate ICD shocks; phenotype subgroups were clearly reported. |
| Miyaji, 2016 | Mixed HCM | Fibrosis imaging (CMR/LGE) | Include | Original HCM CMR study evaluating myocardial fibrosis/LGE with clinically relevant outcomes, including sustained VT/VF and appropriate ICD discharge. |
| Sonnenschein, 2021 | HCM/HOCM subgroup | Fibrosis / extracellular matrix remodeling | Include | Translational study showing fibrosis-related remodeling with increased c-KIT, Collagen 1A1, and MMP2, relevant to adverse myocardial substrate biology. |
| Zhang, 2023 | HOCM | Tissue fibrosis signaling | Include | Pure HOCM tissue study demonstrating collagen deposition and abnormal SMAD2/3/7 signaling, with direct substrate-level relevance to lethal arrhythmia/SCD biology. |
| Marques-Alves, 2025 | Nonobstructive HCM | PET/CMR perfusion, metabolism, fibrosis | Include | Imaging study directly relating fibrosis extension, hypermetabolism, and perfusion abnormalities to SCD risk score in HCM. |
| Cambronero, 2010 | Mixed HCM | Endothelial/microvascular dysfunction biomarker | Include | Original HCM study evaluating von Willebrand factor as a marker of endothelial dysfunction, associated with NSVT and obstruction severity. |
| Liu, 2020 | HOCM | Biomarker with fibrosis/microvascular ischemia relevance | Include | Large HOCM cohort linking cTnI to NSVT, arrhythmia burden, LGE positivity, and estimated 5-year SCD risk. |
| Coleman, 2024 | HCM | Ischaemia, fibrosis, electrophysiologic remodeling | Include | Translational study integrating human patch-clamp data, perfusion CMR, and computational modeling to examine ischaemia-induced arrhythmic risk and sustained re-entry mechanisms. |
| Lin, 2026 | Obstructive HCM | Fibro-inflammatory molecular signaling | Include | Large tissue-based translational study identifying activated fibroblast-derived CRLF1 and LIFR–JAK1/2–STAT3 signaling as mechanistic substrate evidence. |
| Liu, 2023 | Obstructive HCM myectomy tissue | Fibrosis, immune remodeling, spatial transcriptomics | Include | Human myectomy study using single-nucleus RNA-seq and spatial transcriptomics, identifying fibroblast activation, immune remodeling, and fibrosis-related regulatory pathways relevant to arrhythmogenic substrate biology. |
| Shade, 2022 | HCM | Dense and diffuse fibrosis / computational arrhythmogenic substrate modeling | Include | Original human HCM study integrating LGE-CMR, post-contrast T1 mapping, and personalized computational heart modeling; directly evaluates dense and diffuse fibrosis as determinants of ventricular arrhythmia risk and compares performance with conventional clinical SCD risk predictors. |
| Jansen, 2023 | Mixed HCM / genotype-positive cohort | Metabolic biomarker profiling | Exclude | Focused primarily on acylcarnitine-based severity profiling rather than predefined substrate domains directly linked to SCD-related arrhythmic risk. |
| Lin, 2022 | Familial obstructive HCM (3 probands) | miRNA / molecular pathway | Exclude | Extremely small exploratory study without formal analysis of SCD, VT/VF, ICD therapy, NSVT, or other clinically relevant arrhythmic outcomes. |
| Sonnenschein, 2019 | Mixed HCM with HOCM subgroup | circRNA biomarker | Exclude | Focused on phenotype discrimination and severity markers such as LVOT gradient and septal thickness rather than SCD-related arrhythmic outcomes or a defined arrhythmogenic substrate endpoint. |
| Zorzi, 2018 | Non-HCM OHCA cohort | CMR edema/fibrosis after cardiac arrest | Exclude | Not an HCM study; hypertrophic cardiomyopathy was represented only as an isolated diagnosis within a broader OHCA cohort, without extractable HCM-specific analysis. |
| Derda, 2015 | Mixed HCM (HOCM and HNCM) | Circulating miRNA biomarker | Exclude | Biomarker discrimination study without analysis of SCD, VT/VF, ICD therapy, NSVT, or another defined ventricular arrhythmogenic substrate endpoint. |
| Menezes Junior, 2024 | Not applicable | Secondary review / mitochondrial-redox mechanisms | Exclude | Scoping review, not original primary research. |
| Menezes Junior, 2025 | Not applicable | Secondary review / immune-fibrotic remodeling | Exclude | Translational scoping review, not original primary research. |
| Chen, 2024 | Pediatric and adult HCM | Comparative transcriptomics | Exclude | Comparative developmental transcriptomic study without direct analysis of SCD-related arrhythmic outcomes or a defined ventricular arrhythmogenic substrate endpoint. |
| Ma, 2021 | Preclinical mouse HCM model | Gene editing / preclinical molecular therapy | Exclude | Preclinical animal/gene-editing study, not an original human substrate study and without direct SCD-related arrhythmic outcome analysis. |
| Toepfer, 2020 | HCM | Sarcomeric energetic/molecular remodeling | Exclude | Strong mechanistic HCM study, but clinical outcomes centered on heart failure and atrial fibrillation rather than SCD-related ventricular arrhythmic endpoints. |
| Ho, 2017 | Early sarcomeric HCM / mutation carriers | Trial design/protocol | Exclude | Trial design paper without original analyzable outcome data. |
| Marian, 2018 | Mixed HCM | Pilot therapeutic/fibrosis regression trial | Exclude | Pilot randomized HCM trial focused on feasibility and changes in hypertrophy/fibrosis indices without direct analysis of SCD-related ventricular arrhythmic outcomes. |

Abbreviations: HCM = hypertrophic cardiomyopathy; HOCM = hypertrophic obstructive cardiomyopathy; HNCM = hypertrophic nonobstructive cardiomyopathy; CMR = cardiac magnetic resonance; LGE = late gadolinium enhancement; ICD = implantable cardioverter-defibrillator; NSVT = nonsustained ventricular tachycardia; VT/VF = ventricular tachycardia/ventricular fibrillation; SCD = sudden cardiac death; OHCA = out-of-hospital cardiac arrest.

**Supplementary Table S2. Full Search Strategies**

**MEDLINE/PubMed**

("Cardiomyopathy, Hypertrophic"[Mesh] OR "hypertrophic cardiomyopathy"[tiab] OR "hypertrophic obstructive cardiomyopathy"[tiab] OR "obstructive hypertrophic cardiomyopathy"[tiab] OR HCM[tiab] OR HOCM[tiab] OR oHCM[tiab])

AND

("Death, Sudden, Cardiac"[Mesh] OR "sudden cardiac death"[tiab] OR SCD[tiab] OR "sudden death"[tiab] OR "ventricular arrhythmia*"[tiab] OR "ventricular tachycardia"[tiab] OR "ventricular fibrillation"[tiab] OR "non-sustained ventricular tachycardia"[tiab] OR "nonsustained ventricular tachycardia"[tiab] OR NSVT[tiab] OR "implantable cardioverter-defibrillator"[tiab] OR "implantable cardioverter defibrillator"[tiab] OR ICD[tiab] OR "arrhythmogenic substrate"[tiab])

AND

("Fibrosis"[Mesh] OR "Extracellular Matrix"[Mesh] OR fibrosis[tiab] OR fibrotic[tiab] OR "myocardial fibrosis"[tiab] OR "interstitial fibrosis"[tiab] OR "replacement fibrosis"[tiab] OR scar[tiab] OR "myocardial scar"[tiab] OR "extracellular matrix"[tiab] OR ECM[tiab] OR collagen[tiab] OR "matrix remodeling"[tiab] OR "late gadolinium enhancement"[tiab] OR LGE[tiab] OR "microvascular dysfunction"[tiab] OR "coronary microvascular dysfunction"[tiab] OR CMD[tiab] OR "myocardial ischemia"[tiab] OR "myocardial perfusion"[tiab] OR "perfusion reserve"[tiab] OR "myocardial blood flow"[tiab] OR "coronary flow reserve"[tiab] OR inflammation[tiab] OR inflammatory[tiab] OR immune[tiab] OR "immune-inflammatory"[tiab] OR "immune infiltration"[tiab] OR macrophage*[tiab] OR "T cell*"[tiab] OR "T-cell*"[tiab] OR cytokine*[tiab] OR interleukin*[tiab] OR transcriptomic*[tiab] OR transcriptome[tiab] OR "RNA sequencing"[tiab] OR "RNA-seq"[tiab] OR microRNA*[tiab] OR miRNA*[tiab] OR lncRNA*[tiab] OR circRNA*[tiab] OR ceRNA[tiab] OR "competing endogenous RNA"[tiab] OR methyladenosine[tiab] OR m6A[tiab] OR epitranscriptomic*[tiab] OR "hub gene*"[tiab] OR biomarker*[tiab])

AND humans[MeSH Terms]

NOT (review[Publication Type] OR editorial[Publication Type] OR letter[Publication Type] OR comment[Publication Type])

**Embase**

('hypertrophic cardiomyopathy'/exp OR 'hypertrophic cardiomyopathy':ti,ab OR 'hypertrophic obstructive cardiomyopathy':ti,ab OR 'obstructive hypertrophic cardiomyopathy':ti,ab OR hocm:ti,ab OR ohcm:ti,ab)
AND
('sudden cardiac death'/exp OR 'sudden cardiac death':ti,ab OR scd:ti,ab OR 'sudden death':ti,ab OR 'ventricular arrhythmia'/exp OR 'ventricular arrhythmia':ti,ab OR 'ventricular tachycardia'/exp OR 'ventricular tachycardia':ti,ab OR 'ventricular fibrillation'/exp OR 'ventricular fibrillation':ti,ab OR 'nonsustained ventricular tachycardia':ti,ab OR 'non sustained ventricular tachycardia':ti,ab OR nsvt:ti,ab OR 'implantable cardioverter defibrillator'/exp OR 'implantable cardioverter defibrillator':ti,ab OR icd:ti,ab OR 'arrhythmogenic substrate':ti,ab)
AND
('fibrosis'/exp OR 'extracellular matrix'/exp OR fibrosis:ti,ab OR fibrotic:ti,ab OR 'myocardial fibrosis':ti,ab OR 'interstitial fibrosis':ti,ab OR 'replacement fibrosis':ti,ab OR scar:ti,ab OR 'myocardial scar':ti,ab OR 'extracellular matrix':ti,ab OR ecm:ti,ab OR collagen:ti,ab OR 'matrix remodeling':ti,ab OR 'late gadolinium enhancement':ti,ab OR lge:ti,ab OR 'microvascular dysfunction':ti,ab OR 'coronary microvascular dysfunction':ti,ab OR cmd:ti,ab OR 'myocardial ischemia'/exp OR 'myocardial ischemia':ti,ab OR 'myocardial perfusion'/exp OR 'myocardial perfusion':ti,ab OR 'perfusion reserve':ti,ab OR 'myocardial blood flow':ti,ab OR 'coronary flow reserve':ti,ab OR 'inflammation'/exp OR inflammation:ti,ab OR inflammatory:ti,ab OR 'immune response'/exp OR immune:ti,ab OR 'immune inflammatory':ti,ab OR 'immune infiltration':ti,ab OR macrophage'/exp OR macrophage*:ti,ab OR 'T lymphocyte'/exp OR 'T cell':ti,ab OR 'T-cell':ti,ab OR cytokine'/exp OR cytokine*:ti,ab OR interleukin'/exp OR interleukin*:ti,ab OR transcriptomic*:ti,ab OR transcriptome:ti,ab OR 'rna sequencing':ti,ab OR 'rna seq':ti,ab OR microrna'/exp OR microrna*:ti,ab OR mirna*:ti,ab OR lncrna*:ti,ab OR circrna*:ti,ab OR cerna:ti,ab OR 'competing endogenous rna':ti,ab OR methyladenosine:ti,ab OR m6a:ti,ab OR epitranscriptomic*:ti,ab OR 'hub gene':ti,ab OR biomarker'/exp OR biomarker*:ti,ab)
AND [humans]/lim AND [english]/lim
NOT ('review'/it OR 'editorial'/it OR 'letter'/it OR 'note'/it)

**Web of Science Core Collection**

TS=(("hypertrophic cardiomyopathy" OR "hypertrophic obstructive cardiomyopathy" OR "obstructive hypertrophic cardiomyopathy" OR HOCM OR oHCM)
AND
("sudden cardiac death" OR SCD OR "sudden death" OR "ventricular arrhythmia*" OR "ventricular tachycardia" OR "ventricular fibrillation" OR "non-sustained ventricular tachycardia" OR "nonsustained ventricular tachycardia" OR NSVT OR "implantable cardioverter defibrillator" OR ICD OR "arrhythmogenic substrate")
AND
(fibrosis OR fibrotic OR "myocardial fibrosis" OR "interstitial fibrosis" OR "replacement fibrosis" OR scar OR "myocardial scar" OR "extracellular matrix" OR ECM OR collagen OR "matrix remodeling" OR "late gadolinium enhancement" OR LGE OR "microvascular dysfunction" OR "coronary microvascular dysfunction" OR CMD OR "myocardial ischemia" OR "myocardial perfusion" OR "perfusion reserve" OR "myocardial blood flow" OR "coronary flow reserve" OR inflammation OR inflammatory OR immune OR "immune-inflammatory" OR "immune infiltration" OR macrophage* OR "T cell*" OR "T-cell*" OR cytokine* OR interleukin* OR transcriptomic* OR transcriptome OR "RNA sequencing" OR "RNA-seq" OR microRNA* OR miRNA* OR lncRNA* OR circRNA* OR ceRNA OR "competing endogenous RNA" OR methyladenosine OR m6A OR epitranscriptomic* OR biomarker*))
Refined by: DOCUMENT TYPES=(ARTICLE); Languages=(ENGLISH)

**Cochrane Library**

#1 MeSH descriptor: [Cardiomyopathy, Hypertrophic] explode all trees
#2 ("hypertrophic cardiomyopathy" OR "hypertrophic obstructive cardiomyopathy" OR "obstructive hypertrophic cardiomyopathy" OR HOCM OR oHCM):ti,ab,kw
#3 #1 OR #2
#4 MeSH descriptor: [Death, Sudden, Cardiac] explode all trees
#5 ("sudden cardiac death" OR SCD OR "sudden death" OR "ventricular arrhythmia" OR "ventricular arrhythmias" OR "ventricular tachycardia" OR "ventricular fibrillation" OR "non-sustained ventricular tachycardia" OR "nonsustained ventricular tachycardia" OR NSVT OR "implantable cardioverter defibrillator" OR ICD OR "arrhythmogenic substrate"):ti,ab,kw
#6 #4 OR #5
#7 MeSH descriptor: [Fibrosis] explode all trees
#8 MeSH descriptor: [Extracellular Matrix] explode all trees
#9 (fibrosis OR fibrotic OR "myocardial fibrosis" OR "interstitial fibrosis" OR "replacement fibrosis" OR scar OR "myocardial scar" OR "extracellular matrix" OR ECM OR collagen OR "matrix remodeling" OR "late gadolinium enhancement" OR LGE):ti,ab,kw
#10 ("microvascular dysfunction" OR "coronary microvascular dysfunction" OR CMD OR "myocardial ischemia" OR "myocardial perfusion" OR "perfusion reserve" OR "myocardial blood flow" OR "coronary flow reserve"):ti,ab,kw
#11 (inflammation OR inflammatory OR immune OR "immune-inflammatory" OR "immune infiltration" OR macrophage* OR "T cell" OR "T cells" OR "T-cell" OR "T-cells" OR cytokine* OR interleukin*):ti,ab,kw
#12 (transcriptomic* OR transcriptome OR "RNA sequencing" OR "RNA-seq" OR microRNA* OR miRNA* OR lncRNA* OR circRNA* OR ceRNA OR "competing endogenous RNA" OR methyladenosine OR m6A OR epitranscriptomic* OR biomarker*):ti,ab,kw
#13 #7 OR #8 OR #9 OR #10 OR #11 OR #12
#14 #3 AND #6 AND #13

Supplementary Table S3. Methodological Quality and Translational Relevance Assessment of the Included Studies

**Section A. Clinical observational studies**

| **First author, year** | **Study design** | **JBI tool used** | **Key concerns** | **Overall concern** |
| --- | --- | --- | --- | --- |
| Liu, 2020 | Retrospective cohort | JBI Cohort Checklist | Retrospective single-center design; NSVT and estimated SCD risk used instead of hard SCD outcomes | Moderate |
| Miyaji, 2016 | Observational cohort | JBI Cohort Checklist | Mixed HCM cohort; composite endpoint includes arrhythmic and non-arrhythmic events | Moderate |
| Marques-Alves, 2025 | Cross-sectional imaging study | JBI Analytical Cross-Sectional Checklist | SCD risk profile used rather than hard arrhythmic outcomes | Moderate |
| Kunak, 2025 | Retrospective cohort | JBI Cohort Checklist | Composite outcome; indirect biomarker; retrospective design | Moderate to high |
| Cambronero, 2010 | Cross-sectional biomarker study | JBI Analytical Cross-Sectional Checklist | Mixed HCM cohort; NSVT and obstruction severity used as surrogate markers | Moderate |

**Section B. Translational imaging, tissue, molecular, and computational studies**

| **First author, year** | **Study type** | **Population/phenotype definition** | **Exposure/substrate measurement** | **Outcome directness** | **Analytic transparency/validation** | Overall translational concern |
| --- | --- | --- | --- | --- | --- | --- |
| Shade, 2022 | Translational imaging/computational study | Clear adult HCM cohort | Strong LGE + T1-based substrate definition | Moderate to high | Strong computational validation against clinical VA | Moderate |
| Coleman, 2024 | Translational electrophysiologic/computational study | Human HCM-informed datasets | Strong perfusion/electrophysiologic substrate modeling | Moderate | Good analytic framework but model-dependent | Moderate |
| Zhang, 2023 | Tissue-based histopathologic study | Clear HOCM myectomy cohort | Strong tissue fibrosis signaling assessment | Indirect | Acceptable laboratory methods; limited sample | Moderate |
| Lin, 2026 | Tissue-based molecular study | Strong obstructive HCM myectomy cohort | Strong RNA-seq/single-cell/functional validation | Indirect | Strong multimodal validation | Moderate |
| Liu, 2023 | Single-nucleus RNA-seq/spatial transcriptomics study | Clear obstructive HCM myectomy cohort | Strong spatial and transcriptomic substrate definition | Indirect | Strong analytic depth; no direct arrhythmic validation | Moderate |
| Sonnenschein, 2021 | Translational biomarker/tissue study | Acceptable phenotype definition | Moderate ECM/fibrosis-related assessment | Indirect | Acceptable tissue plus in vitro support | Moderate |

Footnote: Clinical observational studies were assessed using design-specific Joanna Briggs Institute (JBI) critical appraisal checklists. Translational imaging, tissue, molecular, spatial transcriptomic, and computational studies were assessed using a prespecified domain-based methodological quality framework because no single validated risk-of-bias instrument adequately captures these designs. Overall translational concern reflects the likely impact of methodologic limitations on interpretation of arrhythmogenic substrate relevance.

**Supplementary Appendix 1. Domain-Based Methodological Quality Framework for Translational Studies**

Because no single validated risk-of-bias instrument adequately captures human translational studies of arrhythmogenic substrate biology in hypertrophic cardiomyopathy, a prespecified domain-based methodological quality framework was developed a priori for this review. This framework was applied to translational imaging, histopathologic, molecular, spatial transcriptomic, and computational studies.

**Domains assessed**

1. Population selection and phenotype definition: assessed whether the study population was clearly defined, whether HCM phenotype assignment was explicit, and whether the cohort was appropriate for the stated biologic question.

2. Adequacy of substrate or exposure measurement: assessed whether fibrosis, ischemia, fibro-inflammatory remodeling, or molecular pathways were measured using technically appropriate and sufficiently described methods.

3. Directness and validity of outcome assessment: assessed whether the study evaluated direct arrhythmic outcomes, validated substrate surrogates, or only indirect mechanistic endpoints.

4. Appropriateness of controls or comparators: assessed whether the study used appropriate comparators, including internal controls, external controls, healthy tissue, or phenotype-stratified comparisons, where applicable.

5. Handling of confounding or biologic heterogeneity: assessed whether major sources of variability, including mixed phenotypes, disease stage, tissue source, and sampling differences, were acknowledged or analytically addressed.

6. Analytic transparency and reproducibility: assessed whether methods were sufficiently described to permit interpretation and whether the analytic workflow was transparent, internally coherent, and reproducible.

7. Validation strategy: assessed the presence or absence of internal validation, external replication, orthogonal validation, or functional experiments supporting the principal findings.

**Domain ratings**

Each domain was rated as low concern, some concern, or high concern.

**Interpretation**

Overall translational concern was determined narratively from the pattern of domain ratings rather than by numeric summation. Studies with strong phenotype definition, robust substrate assessment, and multimodal or functional validation were considered methodologically stronger, even when direct arrhythmic endpoints were absent. Studies relying primarily on indirect biologic inference, limited validation, or poorly defined comparator structures were interpreted more cautiously.

**Use in the review**

These assessments were used to inform interpretation and weighting of the evidence rather than to exclude studies solely on methodological grounds.

**Supplementary Appendix 2. Abbreviations**

- CMR = cardiac magnetic resonance
- CRLF1 = cytokine receptor-like factor 1
- ECM = extracellular matrix
- HCM = hypertrophic cardiomyopathy
- HOCM = hypertrophic obstructive cardiomyopathy
- ICD = implantable cardioverter-defibrillator
- JBI = Joanna Briggs Institute
- LGE = late gadolinium enhancement
- LIFR = leukemia inhibitory factor receptor
- LVOT = left ventricular outflow tract
- MMP2 = matrix metalloproteinase-2
- NSVT = nonsustained ventricular tachycardia
- OHCA = out-of-hospital cardiac arrest
- PET = positron emission tomography
- SCD = sudden cardiac death
- SMAD = small mothers against decapentaplegic
- T1 = longitudinal relaxation time
- TGF-β = transforming growth factor-beta
- VT/VF = ventricular tachycardia/ventricular fibrillation
